# Nutritional status and functional capacity of hospitalized elderly

**DOI:** 10.1186/1475-2891-8-54

**Published:** 2009-11-17

**Authors:** Maria RM Oliveira, Kelly CP Fogaça, Vânia A Leandro-Merhi

**Affiliations:** 1 Institte of Biosciences, UNESP - São Paulo State University, Rubião Junior District, Botucatu-SãoPaulo, Postal Code 18 618-000, MB 510, Brazil; 2 Health Science Faculty, UNIMEP - Methodist University of Piracicaba, Rodovia do Açúcar, Km 153, Piracicaba-São Paulo, Postal Code13 400 911, Brazil; 3 School of Nutrition, PUC-Campinas, São Paulo State, Pontific Catholic University of Campinas, Brazil

## Abstract

**Background:**

The nutritional status of the aging individual results from a complex interaction between personal and environmental factors. A disease influences and is influenced by the nutritional status and the functional capacity of the individual. We asses the relationship between nutritional status and indicators of functional capacity among recently hospitalized elderly in a general hospital.

**Methods:**

A cross-sectional study was done with 240 elderly (women, n = 127 and men, n = 113) hospitalized in a hospital that provides care for the public and private healthcare systems. The nutritional status was classified by the MNA (Mini Nutritional Assessment) into: malnourished, risk of malnutrition and without malnutrition (adequate). The functional autonomy indicators were obtained by the self-reported Instrumental Activity of Daily Living (IADL) and Activity of Daily Living (ADL) questionnaire. The chi-square test was used to compare the proportions and the level of significance was 5%.

**Results:**

Among the assessed elderly, 33.8% were classified as adequate regarding nutritional status; 37.1% were classified as being at risk of malnutrition and 29.1% were classified as malnourished. All the IADL and ADL variables assessed were significantly more deteriorated among the malnourished individuals. Among the ADL variables, eating partial (42.9%) or complete (12.9%) dependence was found in more than half of the malnourished elderly, in 13.4% of those at risk of malnutrition and in 2.5% of those without malnutrition.

**Conclusion:**

There is an interrelationship between the nutritional status of the elderly and reduced functional capacity.

## Introduction

Deterioration of the nutritional status affects and is affected by disease, especially among the elderly [1]. Nutritional diagnosis and the identification of factors that contribute to this diagnosis are, therefore, essential but complex processes. This complexity is due to the occurrence of many changes, both physiological and pathological, which may be taken as inherent to the aging or disease process. However, indirect indicators that likely guarantee proper and healthy eating, such as economic, social, lifestyle and quality of life aspects may represent important tools for assessing nutritional risk [2].

The MNA (Mini Nutritional Assessment) [3] has been an extensively used method to identify risk of malnutrition in the elderly and in those that may benefit from early intervention. The MNA is a simple, low cost and non-invasive method that can be done at bedside [3]. Added MNA scores allow one to screen the elderly who have an adequate nutritional status, those who are at risk of malnutrition and those who are malnourished. The MNA consists of anthropometric and global indicators, including information on eating patterns and self-perception of health, such as: reduced food intake; weight loss of >3 kg body weight; mobility, bed- or chair-bound; psychological stress; neuropsychological problems; body mass index; inability to live independently; taking >3 prescription drugs; having pressure sores or skin ulcers; number of full meals eaten per day; consumption of high-protein foods; consumption of fruits & vegetables; amount of liquids consumed per day; inability to feed self; difficulty in self-feeding; self-view of nutritional status; self-view of health status; mid-arm circumference <21 cm; and calf circumference <31 cm [3]. The tool has been successfully used to assess the nutritional risk of elderly who live independently, receive home care services or are institutionalized, and of patients who are chronically ill, frail, have Alzheimer's disease or cognitive impairment [4]. It has been demonstrated that the sensitivity of this scale is of 96%, the specificity is of 98% and the prognostic value for malnutrition is of 97%^3^. This method has been broadly used among the geriatric population [5-9] and a higher prevalence of malnutrition has been associated with the elderly most in need of care [10].

There are at least 40 screening and assessment tools for subjective nutritional status assessments, and some are for the general population and others for specific populations [11]. The most broadly used of these population-specific tools is the Subjective Global Assessment (SGA), developed by Baker et al in 1982 [3]. The SGA has proven to be one of the most efficient methods to determine nutritional status and make the prognosis of clinical complications [12]. Different from the MNA, the SGA was developed to assess hospitalized individuals, investigating recent weight loss, changes in food consumption, gastrointestinal symptoms, loss of functional capacity, disease-associated stress, and depletion, found upon physical examination [12].

Thus, the SGA focuses mainly on the effect of the disease on nutritional status. When the same population of elderly individuals is assessed by the SGA and MNA, the SGA detects already established malnutrition more precisely, while the MNA detects those who need preventive care [13]. The sensitivities of the SGA and MNA were 93 and 96% respectively, and the specificities were 61 and 26% respectively [13]. The Nutritional Risk Screening (NCR-2002), proposed more recently, has proven to be an important instrument to assess nutritional risk and predict length of hospital stay of elderly patients [14]. Thus, the MNA is considered a very useful instrument for assessing long-term nutritional risk but not as useful for short-term prognoses [15]. Regarding functional autonomy, the MNA considers the mobility of the elderly, if bedbound or wheelchair-bound or if he or she is capable of walking but does not leave the home. The MNA does not assess eating autonomy, that is, if the elderly can prepare his or her own food, if he or she eats without help, if he or she can cut the foods and even if he or she can bring the foods to the mouth.

Functional capacity assessment based on self-reported performance of daily tasks was first assessed by Katz, 1963 [16]. The multidimensional OARS (Older Americans Research Survey) [17] questionnaire was validated and has been used in Brazil [18] for some time now. The questionnaire takes into account the basic activities of daily living (ADL) and the instrumental activities of daily living (IADL). The lack of functional autonomy to look after oneself and to prepare and eat foods is a factor that can result in malnutrition and deserves the attention of professionals and family since functional capacity assessment can be an indicator of nutritional risk which is particularly associated with food intake [19].

The prognosis of elderly inpatients depends not only on the acute physiological conditions inherent to the disease but also on a number of preexisting factors, such as loss of functional independence, loss of cognitive functions, low body weight [20] and corrected arm muscle area [21]. Poor eating habits are predictive of a bad hospitalization prognosis among the elderly [1], suggesting that there is a relationship of interdependence with the other factors. Thus, the objective of this work was to assess the relationship between nutritional status and indicators of functional capacity among recently hospitalized elderly in a general hospital.

## Casuistic and Method

A cross-sectional study was done from September to November 2006 with 240 elderly aging more than 60 years, of both genders (127 womens and 113 mens), hospitalized in a hospital in Piracicaba, SP, Brazil that provides care for the private and public healthcare systems. This study was submitted and approved by the Human Research Ethics Committee of the institution, according to Resolution n° 196/96 of the Brazilian Ministry of Health. The work only began after the patient or caregiver was informed of the purpose of the study and agreed to participate, signing a informed consent form.

All patients aged 60 or more years and who stayed in the hospital for one or more days were included in the study. The lower age limit was chosen according to the second article of the National Policy for the Elderly that classifies individuals aged 60 years or older as elderly [22].

Data collection was done from 24 to 72 hours after admittance through a single interview with the patient or caregiver (if the patient had dementia or some other problem that prevented communication), thus guaranteeing that nearly all the elderly admitted in the studied period were included.

The MNA developed by Guigoz et al.[3] was used for the subjective assessment of the nutritional status. MNA includes questions regarding weight change, dietary change, gastrointestinal symptoms that persist for more than two weeks, functional capacity, physical assessment and disease and its relationship with nutritional requirement. A guidance book was created to calibrate the interviewers before the interviews for data collection to be consistent. In the original MNA version, the Body Mass Index (BMI in weight/height^2^) is included in the assessment. To allow the assessment of bedridden individuals, BMI was substituted by arm circumference (AC) with the patient lying preferably on his or her left side. The agreement of this measurement was determined by the Kappa coefficient (r = 0.89) considering the classification by BMI and AC as follows:

BMI < 19 for AC ≤ P 5

19 ≤ BMI < 21 for P 5 < AC ≤ P 10

21 ≤ BMI < 23 for P 10 < AC < P 85

BMI ≥ 23 for AC ≥ P 85

Where: the percentile (P) for man is P5 = 25 cm, P10 = 26 cm, and P85 = 34 cm, and for woman is P5 = 24 cm, P10 = 25 cm, and P85 = 33 cm [23].

Functional capacity indicators were assessed based on the OARS questionnaire, adapted for the Brazilian population [18]. The present work considered in the set the IADL (using a telephone, walking outside, shopping, meal preparation, housework, self-medicating, handling money) and the ADL (eating, dressing, grooming, walking, transferring, bathing, toileting). They were all considered individually, without worrying about scoring or classifying the degree of autonomy of each participant.

The following items were also investigated: if the elderly lived by him or herself; if he or she had a caregiver (hired or family); if he or she had chronic disease (by verifying the medical record); if he or she was tube fed and if he or she made use of dietary supplements.

The data were analyzed with the elderly divided into groups according to their nutritional status classified by the MNA. The answers to the questionnaire were expressed in numbers and percentages and compared. The proportions were compared by the chi-square test. When the expected values were below 5, two categories were combined (some dependence + complete dependence).

## Results

Among the 240 studied elderly, only 33.8% were classified as having an adequate nutritional status; 37.1% were classified as being at risk of malnutrition and 29.1% were classified as malnourished. Table 1 contains data regarding the variables that correspond to the first MNA phase, showing that all factors were more prevalent among the malnourished individuals. Among these factors, a compromised AC was found in 58.2% of the individuals, which was roughly the proportion found for the other factors (Table 1).

Autonomy to answer the questionnaires was inversely proportional between the elderly classified as malnourished and those at risk of malnutrition or adequately nourished (Table 2). The individuals classified as malnourished presented a higher prevalence of needing a caregiver or tube feeding (Table 2). The use of dietary supplements was lower in these latter two groups (Table 2). Most (85%) of the studied population had a chronic disease and this percentage did not differ among the groups (Table 2). Among the chronic diseases and conditions (one or more conditions in the same individual) of all the assessed individuals, systemic hypertension ranked first (41.7%), followed by diabetes mellitus (29.6%), osteoarticular problems (15.1%), cancer (9.6%), and sequelae of stroke (5.4%). The distribution of chronic diseases and conditions among the malnourished population differed from that of the rest of the sample (*p < 0.001; χ^2 ^= 46.7*). The prevalences were as follows: systemic hypertension, 13.7%; diabetes mellitus, 15.7%; osteoarticular problems, 3.9%; cancer, 12.8%; and sequelae of stroke, 15.7%.

All the IADL and ADL variables assessed were significantly more compromised among the malnourished elderly (Table 3). Among the ADL variables, partial (42.9%) or complete (12.9%) eating dependence is found in more than 50% of the malnourished elderly against 13.4% of those at risk of malnourishment and 2.5% of those adequately nourished.

**Table 1:** Nutritional screening variables of the Mini Nutritional Assessment (MNA), among recently hospitalized elderly patients (N = 240).

Variable	Anwers	M (n = 70)		RM (n = 89)		A (n = 81)		Chi-square
		n	%	n	%	N	%	
Reduced food intake in the last 3 moths	Severe	45	64.2	21	23.6	4	4.9	*p < 0.001*
	Moderate	20	28.6	39	43.8	9	11.1	*χ^2 ^= 118.25*
	Absent	5	7.2	29	32.6	68	84.0	

Weight loss in the last 3 months	More than 3 kg	47	67.2	32	35.9	4	4.9	
	Does not know	10	14.3	18	20.2	2	2.5	*p < 0.005*
	Between 1 and 3 kg	9	12.8	18	20.2	20	24.7	*χ^2 ^= 103.34*
	Absent	4	5.7	21	23.6	55	67.9	

Mobility	Bedbound or wheelchair-bound	37	52.8	9	10.2	6	7.4	*p < 0.001*
	Walks only at home	16	22.8	10	11.2	4	4.9	*χ^2 ^= 80.77*
	Normal	17	24.4	70	78.6	71	87.7	

Stress or acute illness in the last months	Yes No	42 28	60.0 40.0	32 57	35.9 64.1	10 71	12.3 87.7	*p < 0.007* *χ^2 ^= 37.53*

Has neuropsychological problems, dementia or depression	Severe	18	25.8	9	10.2	1	1.25	*p < 0.001*
	Mild dementia	15	21.4	3	3.4	1	1.3	*χ^2 ^= 52.58*
	Absent	37	52.8	77	86.5	79	97.5	

Arm circumference (AC)	≤5	39	58.2	14	15.9	2	2.5	*p < 0.003*
(N = 236)	P5 < AC ≤ P10	0	0	13	14.8	9	11.1	*χ^2 ^= 75.50*
	P10 < AC < P85	25	37.3	45	51.1	49	60.5	
	AC ≥ P85	3	4.5	16	18.2	21	25.9	

M = malnourished; RM = Risk of malnutrition; A = Adequate.

**Table 2:** Variables associated with health and functional autonomy among hospitalized elderly, distributed according to the nutritional status (N = 240).

Variable	Anwers	M (n = 70)		RM (n = 89)		A (n = 81)		Chi-square
		n	%	n	%	N	%	

Interviewed individual	Caregiver	44	62.8	36	40.4	23	28.4	*p = 0.009* *χ^2 ^= 18.55*
		
	User	26	37.2	53	59.6	58	71.6	

Caregiver present	Yes	48	68.6	31	34.8	15	18.5	*p < 0.001* *χ^2 ^= 40.59*
		
	No	22	31.4	58	65.2	66	81.5	

Chronic disease	Yes	60	85.7	83	93.2	76	93.8	*p = 0.148* *χ^2 ^= 3.81*
		
	No	10	14.3	6	6.8	5	6.2	

Tube feeding	Yes	23	32.8	3	3.4	2	2.5	*p = 0.004* *χ^2 ^= 43.9*
		
	No	47	67.2	86	96.6	79	97.5	

Use of supplement	Yes	15	21.4	9	10.1	8	9.9	*p *= 0.06 *χ^2 ^= 5.60*
		
	No	55	78.6	80	89.9	73	90.1	

D = Malnourished; RM = Risk of malnutrition; A = Adequate.

**Table 3:** Functional autonomy for the activities of daily living among hospitalized elderly distributed according to the nutritional status (n = 240).

Variables	Answers	M (n = 70)		RM (n = 89)		A (n = 81)		Chi-square
**Instrumental Activity of daily living (IADL)**		**n**	**%**	**n**	**%**	**n**	**%**	
Phone use	Independence	20	28.6	62	69.7	66	81.5	*p < 0.003*
	Some dependence	17	24.3	20	22.5	10	12.3	*χ^2 ^= 64.40*
	Complete dependence	33	47.1	7	7.9	5	6.2	

Walking outside	Independence	23	32.9	53	59.6	55	67.9	*p < 0.001*
	Some dependence	17	24.3	34	38.2	22	27.2	*χ^2 ^= 41.27*
	Complete dependence	30	42.9	2	2.2	4	4.9	

Shopping	Independence	14	20	49	55.1	55	67.9	*p < 0.001*
	Some dependence	17	24.3	29	32.6	19	23.5	*χ^2 ^= 61.26*
	Complete dependence	39	55.7	11	12.4	7	8.6	

Meal preparation	Independence	17	24.3	44	49.4	60	74.1	*p < 0.005*
	Some dependence	9	12.9	26	29.2	14	17.3	*χ^2 ^= 63.56*
	Complete dependence	44	62.8	19	21.3	7	8.6	

Housework	Independence	14	20	41	46.1	46	56.8	*p < 0.003*
	Some dependence	10	14.3	23	25.8	24	29.6	*χ^2 ^= 45.2*
	Complete dependence	46	65.7	25	28.1	11	13.6	

Self-medicating	Independence	21	30	63	70.8	67	82.7	*p < 0.008*
	Some dependence	20	28.6	19	21.3	10	12.3	*χ^2 ^= 57.7*
	Complete dependence	29	41.4	7	7.9	4	4.9	

Handling money	Independence	14	20	51	57.3	58	71.6	*p < 0.002*
	Some dependence	16	22.8	26	29.2	17	21	*χ^2 ^= 65.33*
	Complete dependence	40	57.2	12	13.5	6	7.4	

**Activity of daily living (ADL)**								
Eating	Independence	31	44.3	77	86.5	79	97.5	*p < 0.001*
	Some dependence	30	42.9	10	11.2	2	2.5	*χ^2 ^= 67.94*
	Complete dependence	9	12.9	2	2.2	0	0	

Dressing	Independence	28	40	80	89.9	77	95.1	*p < 0.002*
	Some dependence	12	17.1	8	9	3	3.7	*χ^2 ^= 88.95*
	Complete dependence	30	42.9	1	1.1	1	1.2	

Grooming	Independence	28	40	79	88.8	79	97.5	*p < 0.001*
	Some dependence	8	11.4	8	9	1	1.2	*χ^2 ^= 94.49*
	Complete dependence	34	48.6	2	2.2	1	1.2	

Walking	Independence	22	31.4	71	79.8	71	87.7	*p < 0.002*
	Some dependence	17	24.3	15	16.9	9	11.1	*χ^2 ^= 83.81*
	Complete dependence	31	44.3	3	3.4	1	1.2	

Transferring	Independence	21	30	73	82	74	91.4	*p < 0.005*
	Some dependence	15	21.4	14	15.7	6	7.4	*χ^2 ^= 74.89*
	Complete dependence	34	48.6	2	2.2	1	1.2	

Bathing	Independence	21	30	73	82	74	91.4	*p < 0.005*
	Some dependence	15	21.4	14	15.7	6	7.4	*χ^2 ^= 74.89*
	Complete dependence	34	48.6	2	2.2	1	1.2	

Toileting	Independence	22	31.4	72	80.9	75	92.6	*p < 0.007*
	Some dependence	16	22.9	15	16.9	6	7.4	*χ^2 ^= 55.79*
	Complete dependence	32	45.7	2	2.2	0	0	

D = Malnourished; RM = Risk of malnourishment; A = Adequate

## Discussion

This study presents data on the outcome of a study that assessed the relationship between nutritional status and functional capacity of hospitalized elderly and there is clearly the need to improve the knowledge on the mechanisms of association between these factors (nutritional and functional states).

In Brazil, deaths associated with malnutrition [24] among the elderly bring to light the discussion on the need to watch this population and intervene nutritionally whenever necessary. In this study, one third of the population being admitted to the hospital was classified by the MNA as being malnourished and the same proportion was classified as being adequately nourished. This reality reinforces the need to invest in assessment and care protocols, especially when dealing with hospitalized patients, where factors such as poor appetite, fatigue, pain and early satiety can reduce oral food intake [25]. Correct intervention helps reduce mortality, improve quality of life and reduce hospitalization costs [25].

This study showed that malnourished individuals are more dependent on others to communicate and meet other needs and they are also more likely to require tube feeding (Table 2) although not forgetting the effect of the disease and the natural aging process. Furthermore, the distribution of the diseases differed among the nutritional status classifications. The malnourished population presented higher proportions of cancer and sequelae of stroke. Cancer is a disease that promotes physiological stress, while stroke mainly compromises functional capacity.

MNA has been broadly used [5-9] to classify the nutritional status and has demonstrated adequate sensitivity and specificity [7]. The elements considered in the screening done in the first phase of the assessment regard a global assessment (reduced food intake, involuntary weight loss, mobility, cognition and body mass) while in phase 2 the dietary habits and self-perception of health are investigated [3]. Low body profile indicators (mass and circumferences) are visible characteristics of protein-calorie malnutrition but good values do not always reflect adequate nutrition. BMI had been recommended as the best anthropometric indicator of nutritional status while arm circumference has not been shown to be a good indicator of nutritional status when used alone [26]. In the validation study which preceded the current study we found a good agreement (r = 0.89) between arm circumference and BMI. This allowed the MNA to be used in bedridden patients where the study was performed.

The global MNA nature allows the inclusion of important factors which do not only classify the nutritional status but also indicate when intervention is necessary to guarantee proper care. Inadequate food intake is the cause of malnutrition while physical and cognitive limitations can prevent adequate food intake [20]. Cereda *et al*., 2008 [27], showed that the poorer functional status was associated with low BMI, sarcopenia and reduced oral intake and the MNA reliably identifies at-risk institutionalised elderly needing higher standards of care, particularly related to eating. Routine documentation of oral intakes and feeding assistance might be useful to prevent weight loss, sarcopenia and functional status deterioration.

The large variability is due to differences in level of dependence and health status among the elderly. In hospital settings, a low MNA score is associated with an increase in mortality, prolonged length of stay and greater likelihood of discharge to nursing homes. Malnutrition is associated with functional and cognitive impairment and difficulties eating. The MNA detects risk of malnutrition before severe change in weight or serum proteins occurs [4].

Functional capacity is interconnected with the quality and quantity of food consumed. The IADL include shopping and preparing meals. In the present study, malnourished individuals were 6 times more dependent on others to shop and prepare meals than those that were adequately nourished (Table 3). Being unable to buy and prepare meals not only interferes with the amount of food ingested but also with the diversity, which may result in boring and unattractive meals. Among the ADL, partial or complete dependence of more than half of the malnourished individuals (Table 3) to eat warn us of the importance to assess the functional capacity while providing nutritional care, as corroborated by the results of a study [28] done with 130 Japanese older than 65 years, where those (48) who totally depended on others to move around were also the ones with the lowest indicators of nutritional status (anthropometry, albumin and food intake).

There is an interrelationship between nutritional and functional statuses. It has already been shown that malnutrition compromises the functional status of the individual [29]. At the same time, functional status impairment increases vulnerability and may affect food consumption negatively [19]. Functional capacity assessment tools have been included in studies that seek to assess nutritional risk [30].

The MNA is a screening and assessment tool with a reliable scale and clearly defined thresholds, usable by health care professionals. It should be included in the geriatric assessment and is proposed in the minimum data set for nutritional interventions^4^. This study reinforces the importance of the MNA as an instrument to assess the nutritional status of the elderly since it represents a global assessment instrument. It also warns us of the need to pay special attention to functional capacity indicators and food intake among the elderly when planning care for this group, especially when they are debilitated by disease.

## Conclusion

A relationship of interdependence between nutritional status and functional status was observed among the studied elderly. Deterioration of the nutritional status was associated with reduced food consumption, recent weight loss, disease-associated stress, degree of self-sufficiency, and functional capacity. The IADL and ADL showed that malnourished elderly were more impaired regarding the activities of daily living, which emphasizes the importance of nutrition. Malnutrition prevalence among the elderly admitted to the hospital was high, probably because of their vulnerability before the disease. Nutritional status deterioration is accompanied by reduced functional capacity. Thus, it is necessary to pay special attention to functional capacity when planning nutritional care for this group, especially when they are debilitated by disease.

## Competing interests

The authors declare that they have no competing interests.

## Authors' contributions

MR carried out the statistical analysis, writing of the article and critically reviewed the article. KC participated in the protocol design and reviewed the manuscript. VA was involved in the protocol and study design, analysis and writing of the article. All authors read and approved the final manuscript.
